# Supplementary material for: Targeting of Human Mitochondrial DNA with Programmable pAgo Nuclease
Source: Cells. 2026 Jan 10;15(2):127. doi: 10.3390/cells15020127 (PMC12839139; doi:10.3390/cells15020127)
Supplement: Supplementary file 1 [file cells-15-00127-s001.zip › cells-4054297-supplementary.pdf]

Table S1. Primer sequences used in this study. List of oligonucleotide primers used for qPCR-based mtDNA quantification (e.g., B2M, D-loop, ND1) and for molecular cloning of constructs (e.g., AmAgo, Su9, FLAG). Sequences are provided in 5′ to 3′ orientation.

Primer	Sequence
B2M_F	TGCTGTCTCCATGTTTGATGTATCT
B2M_R	TCTCTGCTCCCCACCTCTAAGT
D-loop_F	CCTAACACCAGCCTAACCAGATTTC
D-loop_R	AGATTAGTAGTATGGGAGTGGGAGG
ND1_F	CCCTAAAACCCGCCACATCT
ND1_R	GAGCGATGGTGAGAGCTAAGGT
GAG_F	TCTCGACGCAGGACTCG
GAG_R	TACTGACGCTCTCGCACC
AmAgo_Clon_F	AATTCTCGAGATGAATTCGTCAATCTACTTATTTGAATTC
AmAgo_Clon_R	AATTGCTAGCCTATCTGAAATCTCGCGTAATGCTAGG
UbC_F	GCTGAAGCTCCGGTTTTGAACT
WPRE_R	CATAGCGTAAAAGGAGCAACA
Su9_pUltra_clon_F	ACCGGTGCCACCATGGCCTCCACTCGTGTCCT
Su9_pUltra_clon_R	AATGCTAGCATCTCGAGCTTGTCATCGTCATCCTTGTAATC
FLAG_pUltra_clon_F	AATTACCGGTGCCACCATGGACTACAAAGACCATGACGGTGAT

Table S2. Guide RNA sequences used for mtDNA targeting and target DNAs used for in vitro assays.

Name	mtDNA site	Sequence	Comments
**guide RNA**			
ago1	H-strand TAS guide RNA	AGUACAUAAAAACCCAAU	ssRNA guide
ago5	L-strand TAS guide RNA	AUUGGGUUUUUAUGUACU	ssRNA guide
ago6	H-strand TFY guide RNA	ACAUCAUAACAAAAAAUU	ssRNA guide
ago9	L-strand TFY guide RNA	AAUUUUUUGUUAUGAUGU	ssRNA guide
ago10	H-strand LSP guide RNA	AUUUUAUCUUUUGGCGGU	ssRNA guide

ago13	L-strand LSP guide RNA	ACCGCCAAAAGAUAAAAU	ssRNA guide
ago30	L-strand RNR2 guide RNA	AAAGUUAUUUCUAGUUAA	ssRNA guide targeted to ds(mt)DNA region
ago31	no matches guide RNA	AUCAGUAUUCGCGUAUUU	scramble ssRNA guide
**target DNA for in vitro testing**			
ago3	H-strand TAS matrix DNA	CTTGACCACCTGTAGAGTACATAAAAACCCAATCCACATCAAAACCCCCT	target for AmAgo
ago4	L-strand TAS matrix DNA	GGGGTTTTGATGTGGATTGGGTTTTTATGTACTCTACAGGTGGTCAAGTA	target for AmAgo
ago7	H-strand TFY matrix DNA	CCACTTTCCACACAGACATCATAACAAAAAATTTCCACCAAACCCCCCCT	target for AmAgo
ago8	L-strand TFY matrix DNA	GGGGGGTTTGGTGGAAATTTTTTGTTATGATGTCTGTGTGGAAAGTGCT	target for AmAgo
ago11	H-strand LSP matrix DNA	CTAACCAGATTTCAAATTTTATCTTTTGGCGGTATGCACTTTTAACAGTC	target for AmAgo
ago12	L-strand LSP matrix DNA	CTGTTAAAAGTGCATACCGCCAAAAGATAAAATTTGAAATCTGGTTAGGC	target for AmAgo

Table S3. Physicochemical properties of mitochondrial targeting sequences (MTS) fused to AmAgo-WT. Summary of calculated parameters for each MTS-AmAgo-WT construct, including net charge, isoelectric point (pI), and hydropathy index (GRAVY).

Construct	MTS charge	MTS pI	MTS GRAVY	Full charge	Full pI	Full GRAVY
SOD2-3xFLAG-AmAgo-WT	3	10.76	0.163	-2	6.72	-0.336
COX8A-3xFLAG-AmAgo-WT	5	12.48	0.362	0	7.24	-0.322
ATG4D-3xFLAG-AmAgo-WT	10	11.17	-0.337	12	9.06	-0.266
Su9-3xFLAG-AmAgo-WT	13	12.55	-0.272	8	8.82	-0.348

Table S4. Physicochemical properties of mitochondrial targeting sequences (MTS) fused to AmAgo-CD. Summary of calculated parameters for each MTS-AmAgo-CD construct, including net charge, isoelectric point (pI), and hydropathy index (GRAVY).

Construct	MTS charge	MTS pI	MTS GRAVY	Full charge	Full pI	Full GRAVY
SOD2-3xFLAG-AmAgo-CD	3	10.76	0.163	4	8.40	-0.267
COX8A-3xFLAG-AmAgo-CD	5	12.48	0.362	6	8.68	-0.253
ATG4D-3xFLAG-AmAgo-CD	10	11.17	-0.337	7	8.69	-0.338
Su9-3xFLAG-AmAgo-CD	13	12.55	-0.272	10	8.95	-0.331

Figure S1. Assessment of mitochondrial membrane potential by JC-1 staining following AmAgo expression. To assess potential changes in mitochondrial membrane potential following AmAgo expression, cells were stained with the ΔΨm-sensitive dye JC-1. Untreated cells exhibited a typical JC-1 staining pattern with predominant red fluorescence corresponding to J-aggregates, indicative of polarized mitochondria. As expected, treatment with the uncoupler FCCP resulted in a pronounced loss of red fluorescence and a corresponding increase in green JC-1 monomer signal, validating the assay.

Cells expressing AmAgo-WT or the catalytically inactive AmAgo-CD in the absence of guide RNAs displayed JC-1 staining patterns comparable to untreated controls, indicating preserved mitochondrial membrane potential. In contrast, cells expressing AmAgo-WT in the presence of target-specific guide RNAs showed a reduction in JC-1 aggregate (red) signal accompanied by increased green fluorescence, consistent with partial mitochondrial depolarization. No comparable change was observed in cells expressing AmAgo-CD with guide RNAs, indicating that the observed effect depends on the catalytic activity of AmAgo.

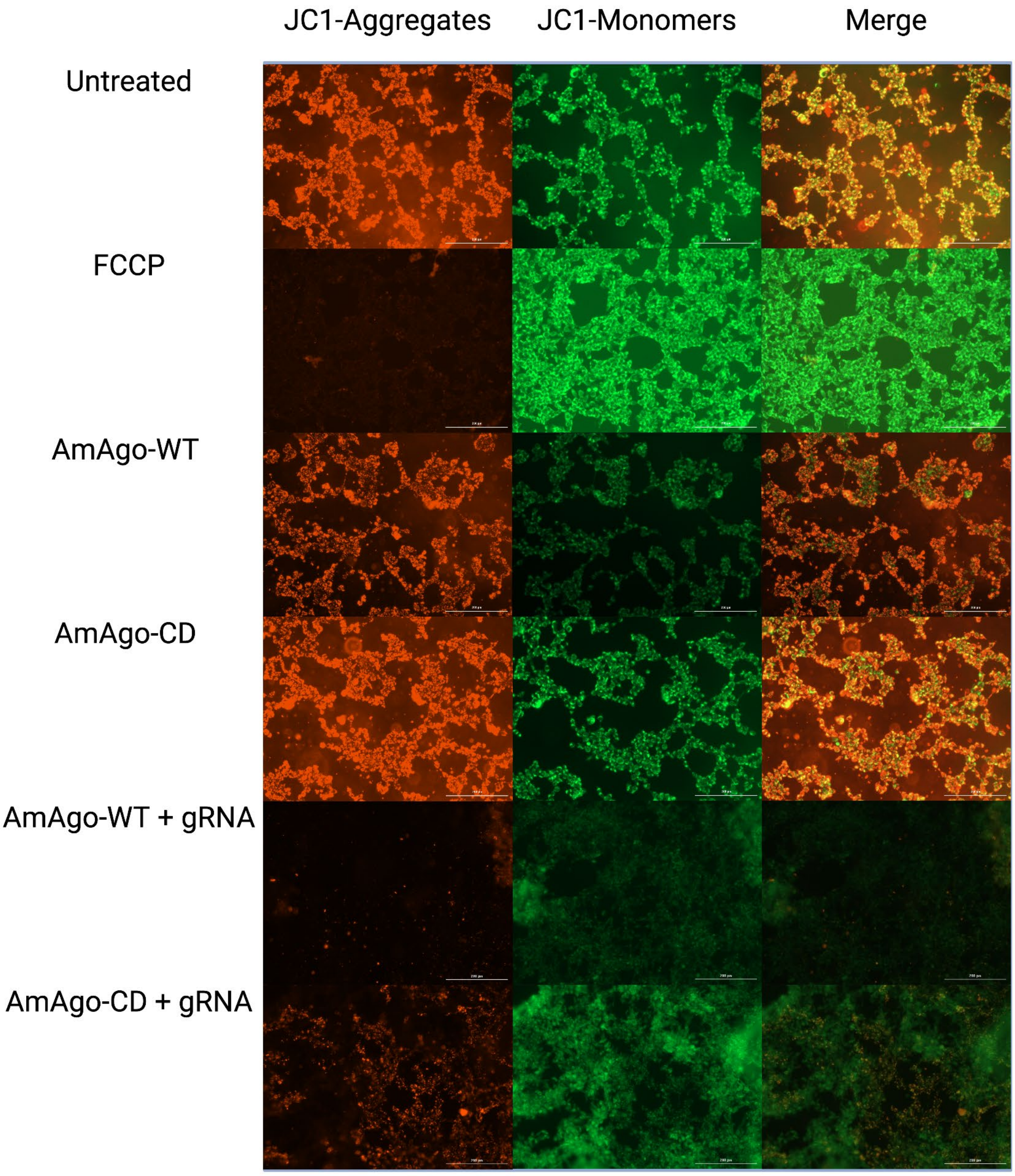

Supplementary Table S5. Comparison of mitochondrial genome editing platforms.
Nuclease sizes and guide lengths are approximate and based on representative constructs reported in the literature. Information on mitoTALENs and mtZFNs was summarized from established studies on protein-based mitochondrial nucleases (e.g., Gammage et al., EMBO Mol Med 2014; Bacman et al., Methods Cell Biol 2020). Data on DdCBE are based on deaminase-based mitochondrial editing systems (Mok et al., Nature 2020). Cas12a-based mitochondrial DNA targeting approaches were summarized from recent reports and reviews (Nikitchina et al., Biochimie 2023; Nikitchina et al., Nucleic Acids Res 2025). Information on Argonaute-based systems is based on the present study and previous work on programmable prokaryotic Argonautes (e.g., Agapov et al., Nucleic Acids Res 2024; Lisitskaya et al., Nucleic Acids Res 2023; Lisitskaya et al., Nature Communication 2022).

Feature	mitoTALENs	mtZFNs	DdCBE	Cas9-based	Cas12a-based	Argonaute-based

				mtDNA tools	mtDNA tools	system (AmAgo)
Targeting principle	Protein–DNA recognition	Protein–DNA recognition	Deaminase-based base editing	Endonuclease-mediated cleavage	Endonuclease-mediated cleavage	RNA-guided ssDNA cleavage
Programmability	Protein engineering	Protein engineering	Protein engineering	RNA-guided	RNA-guided	RNA-guided
Guide molecule	None	None	None	sgRNA	crRNA	Short RNA guide
Typical guide length	—	—	—	~100 nt (sgRNA)	~40–44 nt	~18–24 nt
Nuclease size	~80–115 kDa per monomer (functional dimer ~160–230 kDa)	~50–90 kDa per monomer (design-dependent; functional dimer)	~180–200 kDa (single fusion protein)	~160 kDa (single protein)	~140–150 kDa (single protein)	61.45 kDa (single protein)
Target sequence constraints	High (two binding sites + spacing)	High (two binding sites + spacing)	Context- and window-dependent	PAM-dependent	PAM-dependent	ssDNA accessibility
Primary editing outcome	mtDNA cleavage	mtDNA cleavage	Base conversion (C→T; related platforms enable A→G)	mtDNA cleavage / deletions	mtDNA cleavage / deletions	mtDNA cleavage / depletion
Effect on mtDNA copy number	Transient depletion (~50–70%)	Transient depletion (~50–70%)	Typically preserved	Variable / poorly established	Variable	~70% reduction (this study)
Ability to induce heteroplasmy shift	Yes	Yes	Yes	Potential	Potential	Not addressed (WT mtDNA)
RNA import requirement	No	No	No	Yes	Yes	Yes
Delivery complexity	Large dimeric protein	Dimeric protein	Very large fusion protein	Very large protein–RNA complex	Large protein–RNA complex	Moderate-size protein + short RNA
Reported cytotoxicity	Context-dependent	Context-dependent	Generally low, context-dependent	Variable	Variable	Not observed (this study)
Developmental stage	Established	Established	Rapidly evolving	Experimental / controversial	Experimental	Proof-of-concept
